# Prevalence and correlates for diarrhoea in the mountainous informal settlements of Huye town, Rwanda

**DOI:** 10.1186/2193-1801-3-745

**Published:** 2014-12-16

**Authors:** Dieudonné Uwizeye, Cosmas H Sokoni, Caroline W Kabiru

**Affiliations:** Centre for Environment, Entrepreneurship and Sustainable Development, University of Rwanda, Kigali, Rwanda; Centre for Population Studies and Research, University of Dar es Salaam, Dar es Salaam, Tanzania; Urbanization and wellbeing research program; African Population and Health Research Center, Nairobi, Kenya

**Keywords:** Sanitation status, Diarrhoea, Urban informal settlements, Mountainous environment, Neighbourhoods, Rwanda

## Abstract

Dwellers of urban informal settlements in developing countries are consistently reported to be victims of high diarrhoea prevalence. Studies have frequently reported the association between high diarrhoea prevalence and other factors, such as poor living conditions, inadequate hygiene, and sanitation in these settings. However, little is known about the dynamics of diarrhoea prevalence in mountainous urban informal settlements similar to the Rwandan context. This study was conducted in the Matyazo cell of Huye town to review the prevalence and correlates of diarrhoea. A survey of 214 households and transect walks in the neighbourhoods were conducted during the rainy and dry seasons. Logistic regression was used to analyse the survey data while the thematic analysis technique was used to analyse qualitative data. Results indicated a substantial reduction of diarrhoea prevalence from the rainy to the dry season. It was also found that the prevalence was unequally distributed in the neighbourhoods according to household location. After controlling for other household characteristics and sanitation conditions of around homes, the study indicated that households established at 1800 metres or more above sea level were protected against diarrhoea during both rainy seasons (Adjusted Odds Ratio_ AOR: .42, 95% Confidence Interval_ CI: .13-.81) and dry seasons (AOR: .58, CI: .12-.90) while households found further from the road were likely to suffer from diarrhoea during rainy seasons (AOR: 3.32, CI: 1.47-7.48) as well as in dry seasons (AOR: 1.60, CI: 1.26-4.10). Poor sanitation within and around homes was also found to be associated with the increase of diarrhoea in either season. However, the evidence was not sufficient enough to confirm a significant association between diarrhoea prevalence and other household characteristics. We believe this is due to the strength of environmental factors in mountainous settings.

**Electronic supplementary material:**

The online version of this article (doi:10.1186/2193-1801-3-745) contains supplementary material, which is available to authorized users.

## Introduction

Infectious diseases associated with insufficient supplies of water, poor sanitation and hygiene cause millions of deaths annually. Diarrhoea diseases alone account for around a million deaths globally (Prüss-Üstün and Corvalán, 2006; Prüss et al., 2002; UNICEF, WHO 2009). Different studies report that dwellers of informal settlements are among the most vulnerable population in terms of diarrhoea prevalence due to poor hygienic conditions (Nagdeve, 2002; UN-Habitat, 2011). The characteristics of urban informal neighbourhoods already denote high risks for diarrhoea: overcrowding; poor housing; insecure residential status; limited access to safe water and toilet facilities; poor waste management systems and inadequate drainage systems among others (UN-Habitat, 2007, 2010).

Studies have consistently reported that cities of developing countries were very concerned with the increase of people with poor living conditions in informal neighbourhoods. This results from the exponential growth of cities and towns driven by the influx of rural–urban migrants and the natural population growth amidst weak economies to support the population increase (Jalilian and Weiss, 2000; Kasarda and Crenshaw, 1991; Veary, 2012; Yongsi, 2009). Governments often fail to provide the basic sanitation infrastructure needed to keep up with the population growth. Therefore, residents of urban informal neighbourhoods were forced to mainly rely on contaminated water supplies while waste management systems were inadequate or nonexistent (Karangwa, 2009; Kulabako et al., 2010). Similarly, within East African countries, studies reported that the increase of the urban population was not supported by the provision of an adequate sanitation infrastructure especially in urban informal settlements; thus, exposing the population to high risk for diarrhoea (Godana and Mengiste, 2013; Lubaale and Musyoki, 2011).

In Rwanda, similar issues of inadequate balance between urban population growth and the provision of sanitation infrastructure were reported. Thaxton (2009) as well as Lubaale and Musyoki (2011) observed that dwellers of urban informal neighbourhood usually used unimproved water sources and unsanitary latrine facilities. Drainage and waste management systems were inadequate or nonexistent in some places (Hohne, 2011; Karangwa, 2009; Tsinda and Abbott, 2012). Although the situation is quite similar to other urban informal settlements of developing countries, we assumed that the mountainous nature of the physical environment in Rwanda (REMA, 2010) coupled with poor drainage systems and inadequate waste management may compound the prevalence of diarrhoea and introduce diarrhoea risk variations among dwellers of the same neighbourhood. However, to the best of our knowledge, very few studies have examined the relationship between environmental factors and diarrhoea risk variations in mountainous urban informal settings. Therefore, the ultimate aim of this study is to investigate factors associated with the dynamics of diarrhoea prevalence among household members of all ages living in the mountainous urban informal settlements of Matyazo cell.

### Methodology

#### Selection of the study area

The study targeted towns located in mountainous settings in Rwanda excluding Kigali city as it is currently undergoing very rapid transformation. Six towns of Rwanda fell into our interests namely Huye, Nyamagabe and Muhanga from the Southern Province, Musanze and Gicumbi from the Northern Province, and Gisenyi from Western Province (North-West). Using simple random sampling technique, Huye town was selected as the study setting. At the town level, we were interested in the population living in informal habitat structures since the aim of the study was to investigate how informality of habitat structure interacts with the mountainous physical environment to influence the dynamics of diarrhoea. Therefore, we considered cells where the habitat structure was informal. In Huye town, there were seven such cells: Matyazo of Ngoma sector; Gitwa, Cyarwa, Cyimana and Rango B of Tumba sector; Gatobotobo (Rwabuye) of Mbazi sector; and Rukira cell (Gahenerezo) of Huye sector. The sectors are illustrated on the location map 1, Additional file 1. Using simple random sampling, we selected the Matyazo cell as a study site. In the Rwandan context, according to the decentralisation policy by the ministry of local government (MINALOC), a cell (*akagali*) is the smallest politico-administrative unit and is made of several neighbourhoods (*imidugudu*) (MINALOC, 2012). In this case, Matyazo is made of seven neighbourhoods: Rurenda, Gafurwe, Kamucuzi, Ruvuzo, Rusisiro, Kabeza and Nyabitare. However, the focus of the study was on the entire cell and its residents as one entity rather considering any of the neighbourhoods separately. All the neighbourhoods of Matyazo cell are located on the slopes of Huye Mountain. The altitude varies from approximately 1650 metres high (point taken in the valley) to around 1820 metres high (point taken at the highest level of the hill) above sea level.

#### Study design and sample selection

An ecological study design was used. According to Silva (1999), ecological studies are used to investigate the distribution of a disease in relation to the level of exposure with a focus on a group rather than on individuals. In this study, the basic unit for data collection and analysis was a household rather than individuals.

**Figure 1:**
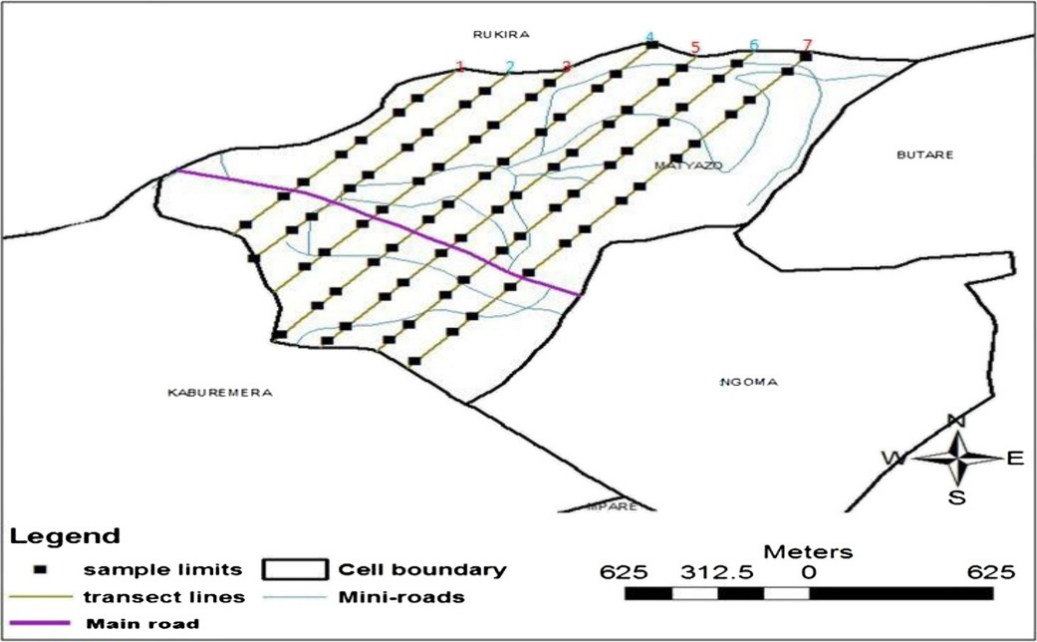
**Sample units on Matyazo cell map.**

It was quite complex to select a representative sample of households in the urban informal habitat structure due to the wide socioeconomic inequalities among residents and informal placement of homes (Nawagamuwa and Viking, 2003; Veary, 2012). In order to overcome the complexities, a strategic spatial random sampling technique was used. This technique, which integrates mapping, was suggested by Veary (2012) in her study in Johannesburg as a more appropriate technique to capture socioeconomic inequalities spatially distributed in urban informal settlements of developing countries. Practically, a digital map of Matyazo cell was used, and seven parallel lines crossing the main road, separated by a hundred and fifty metres were drawn. The lines joined the Northern and Southern border of the cell. Then, equidistant sample units, of one hectare each, were drawn on the lines with reference to the main road. On the field, a GPS (Global Positioning System), a laser distance metre and a compass were used to locate the samples. All the households which fell into the sample unit were enrolled for the survey. The technique gave us twenty six sample units covering a total of 214 households distributed across the study area and included households of various socioeconomic characteristics. The technique is illustrated on Figure 1.

#### Data collection

Data were collected through household surveys and transect walks in the neighbourhoods to observe the status of the physical environment, and learn about the perspectives of the residents on what was being observed. Questions of the survey were answered by the oldest woman among the household members. Women were preferred as the main respondents because, in the context of Rwandan society, they fulfil different roles in the household (wife, mother, family helper, among others) putting them in a position to provide reliable information related to members of the household. During fieldwork, information on the prevalence of diarrhoea within a household was collected with reference to 2 weeks prior to the fieldwork in order to minimise risks for recall bias.

Previous literature indicated that seasonality had a strong effect on diarrhoea prevalence (Alexander et al., 2013; Jadali et al., 2012; Singh et al., 2001). Therefore, we also aimed to investigate the dynamics of diarrhoea prevalence in mountainous settings by season. The fieldwork was conducted in two specific climatic season periods: mid November to mid December (2012) and mid January to mid February (2013), being rainy and dry season periods respectively. We visited the same households for both the first and second fieldwork exercise to investigate the changes of the dynamics of diarrhoea prevalence by season and explored the changes of its correlates. The objective of a one month period between the two data collection periods was to minimise the overlap of the effects of climatic seasons on the prevalence of diarrhoea and the associated factors.

#### Variables and variable measurements

The main dependent variable being analysed in the study was “diarrhoea”. The concept of “diarrhoea” was used with reference to the UNICEF and WHO definition: “having loose or watery stools at least three times per day or more frequently than normal for an individual (UNICEF, WHO 2009, p. 9)”. Respondents were asked to reply “Yes” or “No” to whether any member of their household experienced diarrhoea during the period of interest for the study.

Independent variables, on the other hand, consisted of three groups. First, we considered characteristics of household location within the neighbourhoods: altitude and distance from the main road. Altitude was measured in metres using a GPS while distance from the main road was measured in metres using a laser distance metre. We classified the measured altitude into three categories: (i) 1700 or lower, (ii) 1700–1800 and (iii) above 1800. Similarly, the distance from the main road was classified into three levels: level one (households located within 400 metres from the main road), level two (households located within 400 and 800 metres from the main road), and level three (households located beyond 800 meters from the main road). Second, we considered the status of sanitation within and around homes. We collected data on the presence or absence of stagnant (waste) water, flies and scattered solid waste within and around the household. The third and last group constituted variables which served to explore the household socioeconomic characteristics. We considered four indicators namely the education level of the woman, the observed quality of the home for the family, the type of toilet facility, and the family’s ability to afford a water pipe at the home. To begin with, in light of prior studies, the education of a woman improves the general lifestyle of family members (Masanja et al., 2011) and can contribute to reducing risky practices which lead to diarrhoea (Ogunsola et al., 2013). We, therefore, considered three levels of education for women with reference to the Rwandan educational system: primary education or lower, secondary education level and post-secondary education level. Next, we observed the quality of homes for the households and created three categories (low, medium and good quality homes) reflecting the quality of the main house. Table 1 illustrates the observed characteristics of homes classified into categories.

**Table 1:** **Classification of the quality of the household homes**

Home category	Main description
**Good quality**	Roof of the house covered by new iron sheets; the floor cemented and walls painted; the compound fenced with bricks or cemented (with a gate made of iron material) and the compound floor paved; drainage systems built; generally, the place is regularly maintained to provide comfort to household members.
**Medium quality**	Roof of the main house covered by new iron sheets or new local roofing materials (made of clay); the floor may be cemented and the walls painted but not maintained; the compound fenced by timber and the compound floor not paved but kept in good condition through regular maintenance.
**Poor quality**	The roofing material of the main house is old (either iron sheets or local roofing materials); the floor not cemented and walls not painted; the compound fenced by planted trees or no compound at all; the compound floor neither paved nor sustained.

Equally, we considered the types of toilet facilities as an important indicator for household characteristics considering the objective of the study. With reference to the cost involved to acquire a type of toilet, we devised three categories: traditional pit, fairly improved and modern toilet facilities. According to Sano in Tsinda and Abbott (2012), the construction of a modern toilet facility in Kigali requires 1,500 to 3,000 US dollars while a traditional pit toilet facility can range from 180 to 350 US dollars or less. The cost for each toilet type is based on its quality as was observed: while modern toilet facilities typically satisfy WHO, and UNICEF (2006) standards (flush toilets, or cemented facilities with the pit covered and water connections which allow for a specific place for hand-washing), traditional pit toilet facilities were usually not cemented; instead, they were covered by timber, and doors were unlocked to allow sharing among different neighbouring households whenever needed. This type of toilet facility was not roofed or just loosely roofed, and its walls made of loose materials such as pieces of timber, dry leaves or mud. The entrance was coved by a hanging piece of cloth or a loose door made of timber or a piece of tin. Fairly improved toilet facilities, on the other hand, were generally cemented but the pit not necessarily covered. The entrance was covered with a door carelessly constructed of timber or pieces of tin. However, this type of toilet facility was generally not connected to a water source, and therefore, it did not have a specific place for hand washing after toilet use.

The last variable considered as an indicator for household characteristics was the access to piped water within the homestead. Due to the cost involved to have a water pipe extended to one’s home, (150 US dollars and above according to the distance), households which managed to have piped water extended within their homes were relatively more financially empowered than others.

#### Data management and analysis

Survey data were entered using CSPro 5.0.2 software package (US Census Bureau, 2012) and then transferred to STATA 11 (StataCorp, 2009) for management and analysis. Logistic regression was used to analyse the association between the outcome and the independent variables. Qualitative data from transect walks were in the form of photographs, field notes and records of transcribed interviews. These data were organised using NVivo 9.2 software package (QSR international Pty Ltd, 2010). Analysis of the qualitative data entailed synthesis of data using thematic analyses to provide a robust picture of factors associated with diarrhoea prevalence in Matyazo.

#### Ethics

Ethical clearance was provided by the Research and Ethics Screening Committee (RES-C) of the National University of Rwanda (Ref: DR/0011712012). The head of the household or his/her representative provided written consent through the signing of an informed consent form before completing the household survey.

## Results

### Characteristics of the surveyed households

Tables 2 and 3 indicates that 93.9% of surveyed households were living at an altitude above 1700 metres above sea level and only 6.1% were staying below that altitude. It is also indicated that the concentration of households was high around the main road with 40.6% of the households living within 400 metres from the main road (level one). The concentration reduced within 400 to 800 metres (level two) with 23.8% and rose again beyond 800 metres from the main road (level three) with 35.5% of the surveyed households. Observations within and around homes conducted during the rainy season indicated stagnant waste water among 16.8% of the households. Furthermore, flies were observed within and around 32.2% of the households while solid waste scattered around the physical environment were observed within and around 52.3% of the surveyed households. During the dry season, stagnant waste water was observed around 14% of the households, flies and scattered solid waste were observed within and around 29.4% and 56.1% of the households respectively. Most of the households had low socioeconomic status as it was noticed through the proxy variables. About three quarters of the women (75.7%) had a primary education level or lower and only 4.7% of them had post-secondary education level. Equally, 56.1% were living in low quality homes, 73.8% were using traditional pit toilets, and 74.8% of the surveyed households did not have piped water connections.

**Table 2:** **Environmental and social economic characteristics of households**

Variable	Level	Frequency (n = 214)	%
**Household location**			
***Altitude (in metres)***	1700 or lower	13	6.1
	1700-1800	182	85.0
	Above 1800	19	8.9
***Distance from the road***	Level one	87	40.6
	Level two	51	23.8
	Level three	76	35.5
**Household socioeconomic proxies**			
***Woman’s level of education***	Primary education or lower	162	75.7
	Secondary education	42	19.6
	Post-secondary education	10	4.7
***Home quality***	Low quality	120	56.1
	Medium quality	19	8.9
	High quality	75	35.0
***Types of toilet facility***	Traditional pit toilets	158	73.8
	Fairly improved toilet	43	20.1
	Modern toilet	13	6.1
***Access to piped water***	No piped water at home	160	74.8
	Piped water at home	54	25.2

**Table 3:** **Observed status of the physical environment within and around homes by season**

Variable	Level	Rainy season		Dry season	
		Frequency	%	Frequency	%
***Observed stagnant water***	No	178	83.2	184	86.0
	Yes	36	16.8	30	14.0
***Observed flies***	No	145	67.8	151	70.6
	Yes	69	32.2	63	29.4
***Observed scattered solid waste***	No	102	47.7	94	43.9
	Yes	112	52.3	120	56.1

According to qualitative data, homes were very close to each other around the main roads. The density became lighter further away from the main road and became tighter once again towards the end of the neighbourhoods. The leading reason behind this habitat structure was the high price for plots located near the road and the cost involved to build a house near the road, as community members reported during transect walk:

*Plots near the road are quite expensive. The prices range between two to three million Rwandan francs* (3,000 to 4,500US$)*. It is also not affordable to build near the main road because the masterplan of the town only allows durable construction materials such as bricks and cement, and these are expensive. Therefore, households which cannot afford all those requirements hide themselves behind the others, where town masterplan monitors will not reach very easily. Then, they can build according to what they can afford. […]. Equally, given the costs involved to build a house near the road, the costs for rent for houses near the road are also relatively high that poor households cannot afford* (a male community member aged around 35, met during transect walk)*.*

Equally, it was observed that habitat structures followed the level of altitude as indicated in Table 2. Homes were concentrated at the upper parts of hills leaving the lower parts for forests and bushes. It was observed that due to inadequate waste management and drainage systems, solid waste thrown within the runoff drills were directly drained to lower lands. Sometimes, the runoff overflowed from the usual path and invaded homes and their toilets as most of them were of traditional pit toilet type (Table 2). Subsequently, it was observed that the solid waste scattered at lower land levels consisted of a mixture of household refuse and faeces accumulated in the forests and bushes towards the valleys. Consequently, flies were mostly observed further from the main road where they were following the course of drained waste.

### Diarrhoea risk variations by season

Table 4 shows the estimated diarrhoea risks according to season. It is indicated that the estimated risk for diarrhoea was 55% during the rainy season. When the dry season occurs, the estimated risk reduced by almost a half. From the rainy to the dry season there is a reduction of diarrhoea prevalence by 31%. The results imply that among 1,000 households of Matyazo informal neighbourhoods, 310 extra cases of diarrhoea (approximately one extra case in every three households) would occur during the rainy season compared with the dry season.

**Table 4:** **Estimated diarrhoea risk by season in Matyazo (n = 214)**

	Outcome		Estimated risk (ER)
	***Diarrhoea +***	***Diarrhoea -***	
**Rainy season**	118	96	0.55
**Dry season**	52	162	0.24

During the transect walks, we interviewed residents to understand the community perceptions on the rise of diarrhoea risk during the rainy season. One woman, aged around 60 years, mentioned the issue of drainage systems and substandard toilet facilities which are invaded during the rainy season and brought faeces in contact with people:

*[…] during the December rains, the runoffs invaded our home and collected all types of waste. It even invaded my toilet and those of my neighbours. Waste was brought to surface and drained. I think this contributed to the rise of diarrhoea prevalence we experienced in Matyazo.*

In addition, a male Matyazo resident aged 45 met at level two reflected on the contribution of the status of the physical environment and inability to adapt it:

*The hill slope makes difficult to build the drainage systems for the runoffs. In fact, the rainwater drills accumulates waste and deepen gradually as it rains that we fail to remove the waste. Because of the high altitude, as you can see, waste from the top is accumulated here. That may be one of the reasons we experience diarrhoea during rainy season.*

Another male Matyazo resident aged around 40 met beyond 800 metres from the road reported:

*When it rains, rainwater running off during rainy season collects all the waste from up there to us! It accumulates waste from that school, those churches up there, and the markets places around the road. […] We need adequate drainage system! Not this one drilled by rain water itself. This one keeps stagnant water and waste. Then, our children play with it and flies bring diarrhoea pathogens back to us. Without improved drainage systems we shall always be victims of high diarrhoea prevalence during rainy season.*

According to the community members, lack of adequate drainage and waste management systems in Matyazo cell introduced diarrhoea risk variations among the residents of Matyazo cell although people may have been living in the nearby neighbourhoods.

### Bivariate and multivariable analysis of factors associated with diarrhoea prevalence by season

The results of the bivariate and multivariable analysis indicate that household location and the status of the physical environment within and around the homes are important factors for diarrhoea prevalence. On one hand, Table 5 indicates that households staying at an altitude of 1800 metres plus, above sea level, are less likely to experience an outbreak of diarrhoea during either rainy (OR: .22, 95% CI: .04-.96) and dry seasons (OR: .43, 95% CI: .09-.95) compared with those staying at 1700 metres of altitude or lower. After controlling for other variables, the multivariable analysis indicates that households staying above 1800 metres of altitude are still significantly protected against diarrhoea (AOR: .42, CI: .13-.81 and AOR: .58, CI: .12-.90 during rainy and dry season respectively) compared with those staying at 1700 metres or lower.

**Table 5:** **Bivariate and multivariable analysis of factors associated with diarrhoea prevalence by season**

Factors	Rainy season				Dry season			
	Unadjusted OR	95% CI	Adjusted OR	95% CI	Unadjusted OR	95% CI	Adjusted OR	95% CI
***Household location***								
**Altitude (in metres)**								
1700 & below	1.00		1.00		1.00		1.00	
1700-1800	.37	.10-1.37	.80	.19-3.37	.49	.15-1.59	.62	.16-2.36
1800 & above	.22 *	.04-.96	.42*	.13-.81	.43*	.09-.95	.58*	.12- .90
**Distance from the road (in metres)**								
Level one (400 m)	1.00		1.00		1.00		1.00	
Level two (400-800 m)	3.48*	1.69-7.19	2.96*	1.34-6.57	1.27*	1.05-2.92	1.21*	1.47-3.13
Level three (800 m +)	4.98*	2.55-9.72	3.32*	1.47-7.48	1.79*	1.17-3.68	1.60*	1.26-4.10
***Observed status of sanitation in the physical environment within and around homes***								
**Observed stagnant water**								
No	1.00		1.00		1.00		1.00	
Yes	1.79*	1.04-3.79	1.43	.59-3.49	2.34*	1.10-5.01	1.71	.71- 4.11
**Observed flies**								
No	1.00		1.00		1.00		1.00	
Yes	2.23*	1.22-4.06	1.69*	1.29-2.81	1.79*	1.14-3.42	1.91*	1.28-2.93
**Observed scattered solid waste**								
No	1.00		1.00		1.00		1.00	
Yes	2.11*	1.22-3.67	1.49*	1.07-2.89	3.68*	1.87-7.25	3.59*	1.63 -7.92
***Household socioeconomic proxy variables***								
**Education level of the woman**								
Primary education or lower	1.00		1.00		1.00		1.00	
Secondary education	.35*	.17-.72	.90	.38-2.14	.81	.35-1.82	2.42	.83-7.00
Postsecondary education	.42	.12-1.56	.85	.18-4.02	.74	.15-3.62	2.64	.40-17.26
**Quality of family dwelling**								
Low	1.00		1.00		1.00		1.00	
Medium	.47	.17-1.25	.51	.17-1.52	1.22	.43-3.47	1.77	.56-5.59
High	.63	.35-1.12	.85	.43-1.67	.55	.27-1.13	.68	.30-1.53
**Type of toilet facilities**								
Traditional pit toilets	1.00		1.00		1.00		1.00	
Fairly improved toilets	.48*	.24-.95	1.06	.46-2.44	.61	.26-1.42	.75	.27-2.05
Modern toilets	.41*	.13-.92	1.00	.24-4.12	.22	.03-1.77	.19	.02 -1.90
**Access to piped water**								
Piped water at home	1.00		1.00		1.00		1.00	
No piped at home	3.33*	1.74-6.38	1.96	.89- 4.32	1.84	.83-4.07	1.26	.48 -3.30

*The test indicates a significant at association at 95% level of confidence.

OR: Odds Ratio.

CI: Confidence Interval.

Furthermore, the results suggest an increase of diarrhoea prevalence of nearly 3.5 times during the rainy season (95% CI: 1.69-7.19) among households located at level two distance from the main road (400–800 metres) and almost 5 times (95% CI: 2.55-9.72) among those living at level three (beyond 800 metres), compared with those living at level one (within 400 metres). After controlling for other variables, the adjusted odds ratios decrease slightly for both those living at level two (AOR: 2.96) and level three (AOR: 3.32) compared with those living at level one, but remains statistically significant (95% CI: 1.34 - 6.57 and CI: 1.47-7.48 for level two and three respectively). Similarly, during the dry season, distance from the main road remains a significant variable for diarrhoea prevalence with OR: 1.27 (95% CI: 1.05-2.92) and 1.79 (95% CI: 1.17-3.68) for households living at level two and three respectively at the bivariate analysis and AOR: 1.21 (95% CI: 1.47-3.13) and 1.60 (95% CI: 1.26-4.10) at the multivariable analysis.

Furthermore, results indicate that the poor status of sanitation within and around homes is associated with the prevalence of diarrhoea. The presence of stagnant water within and around homes was independently associated with the prevalence of diarrhoea during both seasons with OR: 1.79, 95% CI: 1.04-3.79 during the rainy season, and OR: 2.34, 95% CI: 1.10-5.01 during the dry season. The presence of flies was also associated with the increase of diarrhoea prevalence in both bivariate analysis (OR: 2.23, 95% CI: 1.22-4.06 during the rainy season and OR: 1.79, 95% CI: 1.14-3.42 during the dry season) and the multivariable analysis (AOR: 1.69, 95% CI: 1.29-2.81 during the rainy season and AOR: 1.91, 95% CI: 1.28-2.93 during the dry season). Similarly, the presence of solid waste scattered within and around homes was associated with the increase of diarrhoea prevalence. The bivariate analysis indicates the odds of 2.11 with 95% CI: 1.22-3.67 and the odds of 3.68 with 95% CI: 1.87-7.25 during rainy and dry seasons respectively, while the multivariable analysis indicates the adjusted odds of 1.49 with 95% CI: 1.07-2.89 and 3.59 with 95% CI: 1.63-7.92 during rainy and dry seasons respectively.

On the other hand, results indicate that improved toilet facilities are independently protective against diarrhoea during rainy seasons. The test shows that households using fairly improved toilet facilities were more than 50% less likely to experience diarrhoea while those using modern toilet facilities were almost 60% less likely to experience diarrhoea compared with those using traditional pit toilets. However, in the multivariable analysis, the association between the types of toilet facilities and the prevalence of diarrhoea within a household was no longer statistically significant. Similarly, the bivariate analysis alone indicates significant association: households with no piped water at home were slightly more than 3 times more likely to experience diarrhoea during rainy seasons (CI: 1.74-6.38) compared with those who had water piped within their homestead.

## Discussion

The results of this study indicated that the mountainous environment introduced variations in diarrhoea prevalence among dwellers of Matyazo cell depending on household location within the neighbourhoods. Both bivariate and multivariable analysis of quantitative data report an enormous increase of diarrhoea risks according to household location in the neighbourhoods during either rainy or dry seasons. Households staying at a high altitude were less likely to experience diarrhoea compared with those staying around the valley. Likewise, the findings of this study indicated that diarrhoea prevalence increases greatly as the distance from the main road increases. In this regard, the study confirmed the assumptions by Hohne (2011) that households staying around drainage openings were more likely to experience diarrhoea in Kigali due to the drills accumulating a mixture of waste carrying diarrhoea pathogens. Qualitative data indicated that these places were mostly at low altitudes where waste was accumulated. The study also complemented the findings by Karangwa (2009) in Huye town that the absence of adequate drainage systems in Huye town exposed households staying at lower lands to high risks for diarrhoea diseases especially during rainy season.

Furthermore, the study indicated that poor environmental sanitation within and around homes was significantly associated with the increase of diarrhoea prevalence among household members. This is consistent with other studies which reported that the poor sanitation status resulting from flooding and inadequate waste management in urban informal settlements are the main environmental factors associated with the rise for diarrhoea diseases in various cities of developing countries (Kulabako et al., 2010; Saha, 2012; UN-Habitat, 2011). Equally, Lubaale and Musyoki (2011) as well as Tsinda and Abbott (2012) observed that poor hygiene and sanitation of the physical environment in Kigali city may contribute to diarrhoea prevalence among poor households living in informal neighbourhoods. On the contrary, the study did not have enough evidence to confirm a significant association between diarrhoea prevalence and other household characteristics considered in the analysis after adjusting for household location in the neighbourhoods and the factors of sanitation in the physical environment. The multivariable analysis of association between the indicators of household characteristics and the prevalence of diarrhoea did not show robust results. We believe that this is due to the strength of the absence of adequate drainage and waste management systems which prevailed because of the mountainous environment.

## Conclusion

This study indicated the incontestable role of unmanaged mountainous environment in the dynamics of diarrhoea prevalence among residents of Matyazo cell located in mountainous settings. The study pioneered research investigating public health issues of diarrhoea prevalence in mountainous urban informal settlements in developing countries. The results underscored the urgency of public investment in providing adequate drainage systems infrastructure in urban informal neighbourhoods located in mountainous settings in order to effectively control the prevalence of diarrhoea. Based on the findings of this study, it is recommended that drainage systems should be created and protected to ensure mountainous settings are viable in the form of towns and cities. The findings of this study have implications for the design of interventions to address diarrhoea in urban informal settlements located in mountainous settings whereby location of households within the neighbourhoods should be particularly considered.

## Electronic supplementary material

Additional file 1: Location map of Huye district and the sectors of interest. (DOC 141 KB)
