# Supplementary figures and images for: Prevalence and correlates for diarrhoea in the mountainous informal settlements of Huye town, Rwanda
Source: Springerplus. 2014 Dec 16;3:745. doi: 10.1186/2193-1801-3-745 (PMC4320130; doi:10.1186/2193-1801-3-745)

**Appendix 1: Location map of Huye district and the sectors of interest**

| 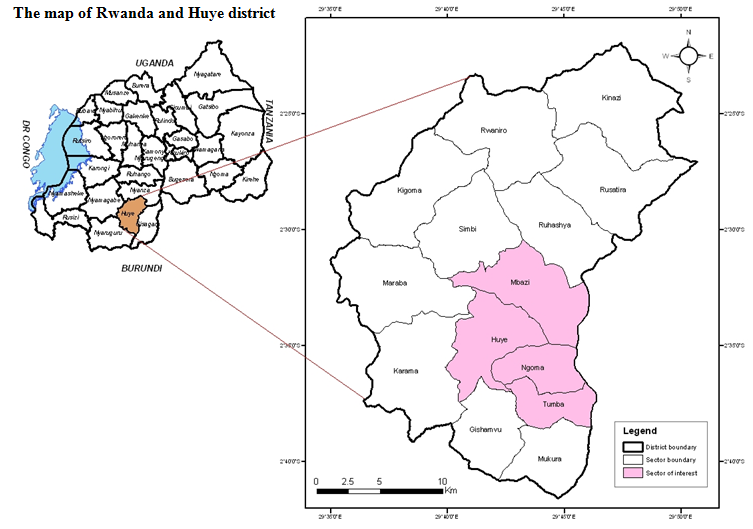 |
| --- |

Supplement: Supplementary file 1 — Additional file 1: Location map of Huye district and the sectors of interest. (DOC 141 KB) [file 40064_2014_1492_MOESM1_ESM.doc]
